# On the G Protein-Coupled Receptor Neuromodulation of the Claustrum

**DOI:** 10.1007/s11064-019-02822-4

**Published:** 2019-06-06

**Authors:** Dasiel O. Borroto-Escuela, Kjell Fuxe

**Affiliations:** 1 Department of Neuroscience, Karolinska Institutet, Retzius väg 8, 17177 Stockholm, Sweden; 2 Department of Biomolecular Science, Section of Physiology, University of Urbino, Campus Scientifico Enrico Mattei, via Ca’ le Suore 2, 61029 Urbino, Italy; 3 Observatorio Cubano de Neurociencias, Grupo Bohío-Estudio, Zayas 50, 62100 Yaguajay, Cuba; 4 Biomedicum, Solnavagen 9, 17177 Stockholm, Sweden

**Keywords:** Claustrum, Volume transmission, Dopamine receptor, Opioid receptor, Heteroreceptor complexes, Receptor–receptor interactions

## Abstract

G protein-coupled receptors modulate the synaptic glutamate and GABA transmission of the claustrum. The work focused on the transmitter–receptor relationships in the claustral catecholamine system and receptor–receptor interactions between kappa opioid receptors (KOR) and SomatostatinR2 (SSTR2) in claustrum. Methods used involved immunohistochemistry and in situ proximity ligation assay (PLA) using confocal microscopy. Double immunolabeling studies on dopamine (DA) D1 receptor (D1R) and tyrosine hydroxylase (TH) immunoreactivities (IR) demonstrated that D1R IR existed in almost all claustral and dorsal endopiriform nucleus (DEn) nerve cell bodies, known as glutamate projection neurons, and D4R IR in large numbers of nerve cell bodies of the claustrum and DEn. However, only a low to moderate density of TH IR nerve terminals was observed in the DEn versus de few scattered TH IR terminals found in the claustrum. These results indicated that DA D1R and D4R transmission in the rat operated via long distance DA volume transmission in the rat claustrum and DEn to modulate claustral-sensory cortical glutamate transmission. Large numbers of these glutamate projection neurons also expressed KOR and SSTR2 which formed KOR-SSTR2 heteroreceptor complexes using PLA. Such receptor–receptor interactions can finetune the activity of the glutamate claustral-sensory cortex projections from inhibition to enhancement of their sensory cortex signaling. This can give the sensory cortical regions significant help in deciding on the salience to be given to various incoming sensory stimuli.

## Introduction

Claustrum has become a brain region of high interest by the introduction of the hypothesis by Crick and Koch that claustrum has a major role in consciousness [1], a view further developed by Smythies et al. [2].

The Golgi type 1 neurons with medium-sized nerve cell bodies build up the major population of neurons in the claustrum [3]. They represent projection neurons with spiny dendrites and appear to connect to all cortical regions in a reciprocal way, especially on the ipsilateral side [4, 5]. They are excitatory and operate via glutamate transmission. Also a significant population of small spiny inhibitory GABA interneurons exist in the claustrum with axons that ramify within the claustrum and exert an inhibitory synaptic regulation of the glutamate projection neurons. The Golgi type 1 neurons and the small spiny inhibitory GABA interneurons come together and form the claustrum without any signs of organization in terms of lamellation and is regarded as a subcortical structure.

Ventrally to the claustrum another subcortical structure is located, the dorsal endopiriform nucleus (DEn) [6]. It is regarded as mainly reciprocally connected with the olfactory cortex and is proportionally highly developed in rodents.

In recent years it has become of interest to know how G protein-coupled receptors (GPCRs) can modulate the synaptic glutamate and GABA transmission of the claustrum including also the DEn. It involves the use of the concepts of volume transmission and receptor–receptor interactions in GPCR heteroreceptor complexes [7–10]. In view of the role of dopamine (DA) transmission in reward and salience of sensory inputs it becomes of special interest to study the tyrosine hydroxylase (TH) immunoreactive (IR) nerve terminal networks and the DA receptor subtype D1R, D2R and D4R location in the claustrum, the DEn and their surroundings, especially the insula cortex. The cortical DA innervation originates from the ventral tegmental area and/or the substantia nigra [11].

So far TH IR nerve terminal networks were found in the DEn but not in claustrum in the rat [12] while they were demonstrated in the claustrum of the pig [13]. However, D1R with a high turnover was found in rat forebrain regions including the claustrum [14] and a moderate density of D1R was demonstrated in rat claustrum and dorsal endopiriform cortex [15]. D2R mRNA levels were also observed in the claustrum and the DEn of the rat [16].

Furthermore, the highest density of kappa opioid receptors (KOR) is present in the claustrum and KOR agonists besides reducing pain can produce hallucinations and dissociative effects [17]. It is of interest that a psychoactive compound salivinorin A can produce synaesthesia by activation of KOR [18]. This is in line with the view that claustrum participates in consciousness [1, 6]. We will therefore also study the KOR IR location in the claustrum and DEn and its possible colocation with somatostatin receptor 2 (SSRT2), also enriched in these nuclei as demonstrated with in situ hybridization methods [19]. In view of the demonstration of heterodimerization of SSRT2 and MOR in HEK293 cells [20] it also becomes of substantial interest to study if KOR-SSRT2 heteroreceptor complexes exist in the claustrum using the proximity ligation assay [21–23]. The major aim is to understand the modulation of the projection neurons of the claustrum by GPCRs and their receptor complexes [5, 6].

## Material and Methods

### Animals

All experiments were performed using 3–4 months old male Sprague–Dawley rats (SD) (Scanbur, Sweden). The animals were group-housed under standard laboratory conditions (20–22 °C, 50–60% humidity), with food and water available ad libitum. All procedures were conducted in accordance with the Stockholm North Ethical Committee of Animal Experimentation, the Swedish National Board for Laboratory Animals (NBLA) and European Community’s Council Directive (2010/63/EU) guidelines for accommodation and care of animals.

### Tissue Preparation

Animals were deeply anesthetized with pentobarbital and perfused intracardially with 4% paraformaldehyde in saline. Brains were dissected out and immersed in the same fixative overnight, in 10% sucrose for 24 h and in 30% sucrose for 2 weeks. Coronal sections (35 μm, Bregma 1.60–1.70 mm) were cut on a cryostat and immersed in Hoffman solution [23] for free-floating immunohistochemistry.

### Immunohistochemistry

Sections were treated with 10 mM citrate (pH 6) of 65 °C at room temperature (RT) for 30 min. After three washes with PBS (5 min each), 10 mM glycine (in PBS) was applied for 20 min, followed by two washes with PBS (5 min). Sections were then permeabilized with 0.3% Triton X-100 for 30 min, washed twice with PBS (5 min) and pre-incubated in blocking buffer (SuperBlock®, ThermoFisher Scientific Prod# 37,515) for 30 min. Subsequently, they were incubated under gentle agitation at 4 °C overnight with each of the following primary antibodies: mouse monoclonal anti-KOR (1:25, 1:50) (ab201552, abcam), anti-Tyrosine hydroxylase Alexa Fluor 488 (1:100) (MAB318-af488, Millipore), rabbit anti-D4R (orb39453, Biorbyt), rabbit anti-D1R (orb107494, Biorbyt), mouse anti-D2R (1:600) (MABN53, Millipore) and rabbit monoclonal anti-SSTR2 (1:100) (ab134252, abcam). After two washes (5 min) with 1:2 dilution of blocking buffer in PBS, slices were incubated for 2 h at 37 °C with the secondary antibodies Alexa Fluor 488-conjugated goat anti-mouse (A11001) or anti-rabbit (A11034, A11008) (1:200, Invitrogen). Following two washes with the diluted blocking buffer in the dark, sections were mounted on SuperFrost glass slides with the addition of mounting medium (Duolink® In Situ Mounting Medium with DAPI, DUO82040, Sigma-Aldrich) and stored at − 20 °C.

### *In situ Proximity Ligation Assay (*in situ* PLA)*

To study the existence of KOR-SSTR2 heteroreceptor complex in the claustrum and DEn the in situ PLA was performed as described previously [21–24]. Free-floating formalin fixed brain sections (35 μm-thick, cut using a cryostat) at Bregma level ( + 1.0 mm) from rats were employed using the following primary antibodies: mouse monoclonal anti-KOR (ab201552, abcam) and rabbit monoclonal anti-SSTR2 (ab134252, abcam). Control experiments employed only one primary antibody or cells transfected with cDNAs encoding only one type of receptor. The PLA signal was visualized by using a Leica TCS-SL SP5 confocal microscope (Leica, USA) and the Duolink Image Tool software. Briefly, fixed free-floating rat brain sections (storage at − 20 °C in Hoffman solution) were washed four times with PBS and quenched with 10 mM glycine buffer, for 20 min at room temperature. Then, after three PBS washes, incubation took place with a permeabilization buffer (10% fetal bovine serum (FBS) and 0.5% Triton X-100 or Tween 20 in Tris buffer saline (TBS), pH 7.4) for 30 min at room temperature. Again the sections were washed twice, 5 min each, with PBS at room temperature and incubated with the blocking buffer (0.2% BSA in PBS) for 30 min at room temperature. The brain sections were then incubated with the primary antibodies diluted in a suitable concentration in the blocking solution for 1–2 h at 37 °C or at 4 °C overnight. The day after, the sections were washed twice, and the proximity probe mixture (affinity purified donkey anti-mouse MINUS (DUO92004) and anti-rabbit PLUS (DUO92002) (Duolink® PLA probes, Sigma-Aldrich)) was applied to the sample and incubated for 1 h at 37 °C in a humidity chamber. The unbound proximity probes were removed by washing the slides twice, 5 min each time, with blocking solution at room temperature under gentle agitation. The sections were incubated with the hybridization-ligation solution (BSA (250 g/ml), T4 DNA ligase (final concentration of 0.05 U/µl), Tween-20 (0.05%), NaCl 250 mM, ATP 1 mM and the circularization or connector oligonucleotides (125–250 nM)) and incubated in a humidity chamber at 37 °C for 30 min. The excess of connector oligonucleotides was removed by washing twice, for 5 min each, with the washing buffer A (Sigma-Aldrich, Duolink Buffer A (8.8 g NaCl, 1.2 g Tris Base, 0.5 ml Tween 20. Dissolved in 800 ml high purity water, pH to 7.4) at room temperature under gentle agitation and the rolling circle amplification buffer was added to the sections and incubated in a humidity chamber for 100 min at 37 °C. Then, the sections were incubated with the detection solution through hybridization (fluorescent oligonucleotide probes) in a humidity chamber at 37 °C for 30 min. In a last step, the sections were washed twice in the dark, for 10 min each, with the washing buffer B (Sigma-Aldrich, Duolink Buffer B (5.84 g NaCl, 4.24 g Tris Base, 26.0 g Tris–HCl. Dissolved in 500 ml high purity water, pH 7.5) at room temperature under gentle agitation. The free-floating sections were put on a microscope slide and a drop of appropriate mounting medium containing DAPI giving a blue staining of the nuclei (e.g., VectaShield or Dako) was applied. The cover slip was placed on the section and sealed with nail polish. The sections were protected against light and stored for several days at − 20 °C before confocal microscope analysis.

### Analysis

The confocal microscope Leica TCS-SL (Leica, USA) was used. The number of samples (n) in each experimental condition is indicated in figure legends. The claustrum was pinpointed as previously described anatomically in coronal sections at the Bregma level ( + 1.00 mm).

## Results

### Immunohistochemistry

#### Dopamine D1R, D2R and D4R IRs and Their Relationship to Tyrosine Hydroxylase (TH) IR Nerve Terminal Networks.

##### D1Rs

**Fig. 1:**
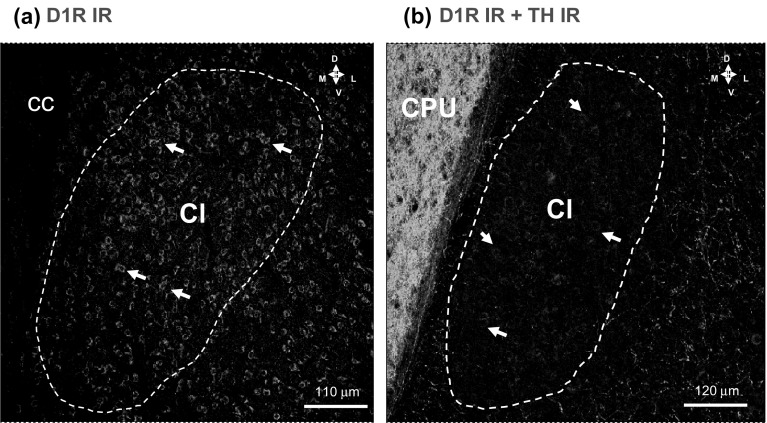
Illustration of the D1R and tyrosine hydroxylase (TH) single and double immunofluorescence studies in the rat claustrum. Microphotographs from coronal sections of rat brain at Bregma ( + 1.00 mm) level. **a** A high density of D1R IR nerve cell bodies (in green) is observed using single immunolabeling within the claustrum and in the deep layers of the insular cortex ventrolateral to the Cl. Images are representative for the three rats studied. Scale bar is shown. **b** A high density of D1R IR nerve cell bodies and proximal dendrites (in red) and a very low density of tyrosine hydroxylase IR nerve terminals (TH) (in green) are observed using double immunolabeling. They do not codistribute within the claustrum. The TH IR nerve terminal plexa exist in high densities laterally to the Cl in the deep layers of the insular cortex and in very high densities in CPU, medial to the Cl. As indicated with white arrows, the D1R IR is mainly limited to the nerve cell bodies with only a few proximal dendrites labeled. Images are representative for the three rats studied. Scale bar is shown. *Cl* Claustrum, *CPU* Caudate putamen, *CC* corpus callosum (Color figure online)

In double immunolabeling studies on D1R and TH IR, D1 IR nerve cell bodies in red were found all over the claustrum with only few TH IR nerve terminals in green present (Fig. 1b). High densities of TH IR nerve terminals surrounded the claustrum on the lateral side located in the deep layers of the insula cortex. The high density of oval to round, medium-sized D1R IR nerve cell bodies in claustrum were more clearly observed when visualized with a green fluorescence with lack of lamination or other types of orientation (Fig. 1a). The D1R IR is mainly limited to the nerve cell bodies with only a few proximal dendrites labeled. This becomes even more clear in a high magnification (data not shown).

**Fig. 2:**
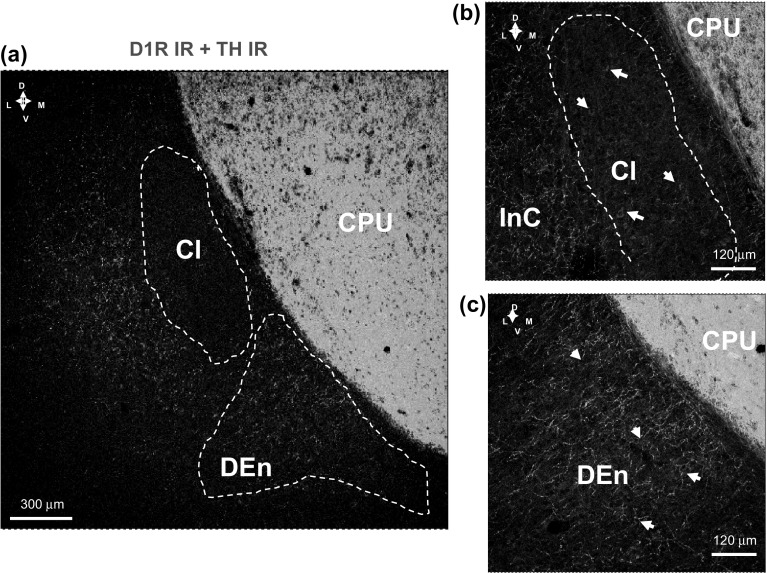
Illustration of the D1R (in red) and tyrosine hydroxylase (TH) (in green) double immunofluorescence studies in the claustrum, caudate putamen and insular layers of the rat brain. Microphotographs from coronal sections of rat brain at Bregma ( + 1.00 mm) level. **a** A high density of D1R IR nerve cell bodies and proximal dendrites (in red) and a high density of tyrosine hydroxylase (TH) IR nerve terminal plexa (in green) are observed and shown to co-distribute within the insular layers (InC) and the dorsal ndopiriform nucleus (DEn) but not within the Cl. A very high density of TH IR nerve terminals is observed in the CPU. **b** The relationship of the Cl to the InC is given. With white arrows are indicated some of the D1R IR nerve cell bodies and proximal dendrites (in red). Images are representative for the three rats studied. **c** A high density of D1R IR nerve cell bodies and proximal dendrites (in red) and a high density of tyrosine hydroxylase IR nerve terminal plexa (TH) (in green) are observed and shown to codistribute within the DEn (for some examples, see white arrows). Images are representative for the three rats studied. Scale bar is shown. *DEn* dorsal endopiriform nucleus, *CPU* caudate putamen, *Cl* claustrum, *InC* insular cortex (Color figure online)

Instead double immunolabeling of TH and D1R IR in the DEn demonstrated a moderate density of TH IR terminals innervating the medium sized D1R positive cell bodies of the DEn (Fig. 2a–c). A moderate to high density of TH IR nerve terminal networks was found in layers II–VI of the insular cortex matched by high densities of D1R IR nerve cell bodies in these layers (Fig. 2a, b). A very high density of TH IR terminals was found in the caudate putamen (Fig. 2a–c).

##### D2Rs

**Fig. 3:**
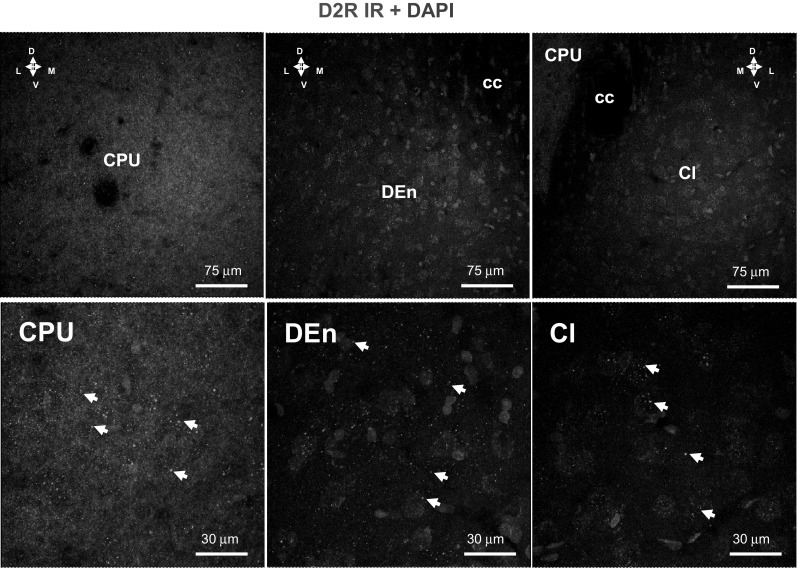
Illustration of the D2R (in green) single immunofluorescence studies in the caudate putamen (CPU), claustrum (Cl) and in the dorsal endopiriform nucleus (DEn) of the rat brain. Microphotographs from coronal sections of rat brain at Bregma ( + 1.00 mm) level. A low density of punctate D2R IR (in green), probably located in distal dendrites, is observed together with a few weakly to moderately D2R IR nerve cell bodies within the claustrum and in the DEn where a higher density of punctate D2R IR were found in the caudate putamen. Images are representative for the three rats studied. The nuclei are shown in blue by DAPI. Scale bars are shown. *Cl* Claustrum, *CC* corpus callosum, *DEn* dorsal endopiriform nucleus, *CPU* caudate putamen (Color figure online)

In contrast to D1R IR, only weak punctate D2 IR of low density was found in the neuropil of the claustrum and DEn while high densities of strong punctate D2 IR was found in the neuropil of caudate putamen which is well-known from previous work [25, 26] (Fig. 3). Scattered weakly D2R IR nerve cell bodies were also found in the claustrum and the DEn (Fig. 3).

##### D4Rs

**Fig. 4:**
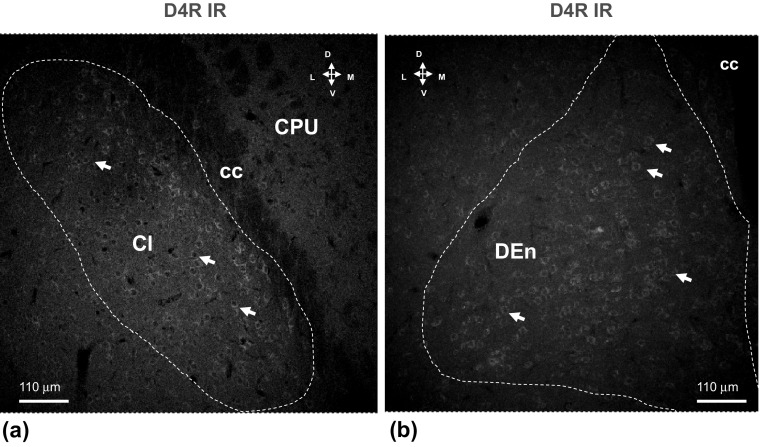
Illustration of the D4R (in green) single immunofluorescence studies in the claustrum (**a**) and in the dorsal endopiriform nucleus (**b**) of the rat brain. Microphotographs from coronal sections of rat brain at Bregma ( + 1.00 mm) level. A medium density of D4R IR nerve cell bodies and proximal dendrites with moderate to high IR (in green) is observed within the claustrum (**a**) and in the DEn (**b**). D4R IR is also observed in CPU but with a more diffuse appearance. Images are representative for the three rats studied. Scale bars are shown. *Cl* Claustrum, *CC* corpus callosum, *DEn* dorsal endopiriform nucleus (Color figure online)

In claustrum a medium density of moderately D4 IR nerve cell bodies were observed with highest densities along the medial surface towards the crus cerebri (Fig. 4). In higher resolution clusters of high densities of D4R IR nerve cell bodies are observed in this part of the claustrum (data not shown). In the DEn ventral to the claustrum also clusters of D4R IR nerve cell bodies could be identified, in this case mainly in a ventrolateral position (Fig. 4).

#### KOR and SSRT2 IRs

##### KOR

**Fig. 5:**
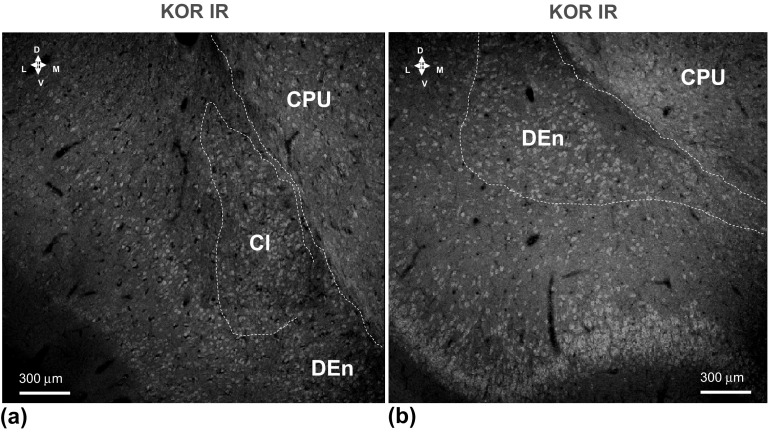
Illustration of the KOR (in green) single immunofluorescence studies in the claustrum (**a**) and in the dorsal endopiriform nucleus (**b**) of the rat brain. Microphotographs from coronal sections of rat brain at Bregma ( + 1.00 mm) level. A very high density of strongly KOR IR nerve cell bodies (in green) is observed within the claustrum (**a**) and in the DEn (**b**). A moderate to high density is observed in the CPU (**a**, **b**) and a high to very high density in the piriform cortex (**b**). Images are representative for the three rats studied. Scale bars are shown. *Cl* Claustrum, *CC* corpus callosum, *DEn* dorsal endopiriform nucleus (Color figure online)

The majority of nerve cell bodies in the Claustrum were found to be strongly KOR immunoreactive (Fig. 5 a), which was true also for the nerve cell bodies in the deeper layers of the insula cortex. Similar results on KOR IR were obtained in the DEn (Fig. 5 b).

##### SSRT2

**Fig. 6:**
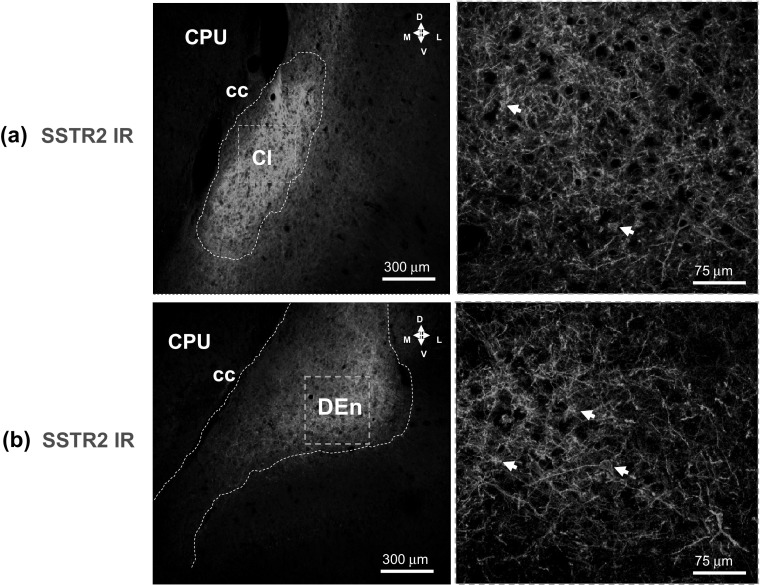
Illustration of the SSTR2 (in green) single immunofluorescence studies in the claustrum (**a**) and in the dorsal endopiriform nucleus (**b**) of the rat brain. Microphotographs from coronal sections of rat brain at Bregma ( + 1.00 mm) level. A very high density of SSTR2 IR distal dendritic plexa of high intensity (in green) is observed within the claustrum (**a**) and in the DEn (**b**). With white arrows are point out the SSTR2 IR found in the cell bodies. In low magnification only a strong diffuse SSTR2 immunofluorescence is found. Images are representative for the three rats studied. Scale bars are shown. *Cl* Claustrum, *CC* corpus callosum, *DEn* dorsal endopiriform nucleus (Color figure online)

A strong diffuse SSRT2 IR was observed all over the claustrum, especially in the lateral position, and in the DEn, especially the ventrolateral position (Fig. 6a, b). In higher magnifications it was found that the strong SSRT2 IR was mainly located to the dendritic plexus of these regions but also in the nerve cell bodies (Fig. 6a, b).

#### Proximity Ligation Assay (PLA)

**Fig. 7:**
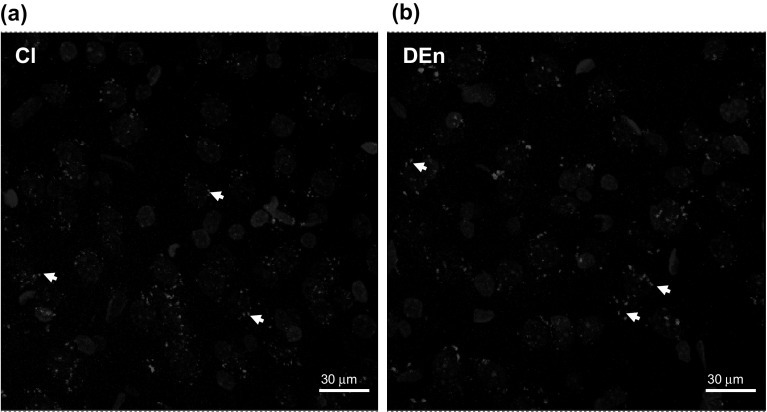
Illustration of the KOR-SSTR2 heteroreceptor complex in the claustrum (**a**) and in the dorsal endopiriform nucleus (**b**) of the rat brain. Microphotographs are taken from a coronal section of rat brain at Bregma (± 1.00 mm) level. The existence and distribution of the KOR-SSTR2 heteroreceptor complexes in the claustrum (**a**) and in the DEn (**b**) are demonstrated using the in situ proximity ligation assay (in situ PLA) technique. They are visualized as red PLA blobs (clusters) found in relation to a large number of cells using confocal laser microscopy. The nuclei are shown in blue by DAPI. Pictures were taken as multi z-scan (20, 1 μm each). *Cl* Claustrum, *DEn* dorsal endopiriform nucleus (Color figure online)

In these experiments it became possible to demonstrate that specific PLA positive clusters could be formed between KOR and SSRT2 GPCRs in the claustrum and the DEn (Fig. 7a, b).

## Discussion

The current findings help to understand the transmitter-receptor relationship in DA transmission taking place in the claustrum. We could validate an almost complete lack of TH IR nerve terminals in the claustrum of the rat as previously observed [12]. However, scattered TH IR nerve terminals were found in this region representing catecholamine nerve terminals. In contrast, a moderate to high density of TH IR nerve terminals were present in the deep layers of the insula cortex closely surrounding the lateral border of the claustrum. The claustrum contained a high density of strongly D1R IR nerve cells in a perisomatic position, a medium density of moderately D4R IR nerve cell bodies, especially along the medial border and a few clusters of weakly D2 IR nerve cell bodies. In line with previous work demonstrating transmitter–receptor mismatches in e.g., nucleus accumbens shell and intercalated islands of the amygdala [25, 27–30] we propose that slow long distance DA volume transmission (VT) in the order of 50–100 um can be the transmission involved in the claustrum of the rat. The diffusion and flow of DA may be facilitated by a special angioarchitecture of this frontal region that may make the arterial pulse especially effective [31] in enhancing flow in the perivascular pathways of the G lymphatic system [32–34]. Possible existence of uncoupling protein 2/3 IR in the TH IR terminal rich area of the surrounding regions may also be relevant since local heat gradients may be produced increasing flow of DA into the claustrum [30]. In addition, a reduced tortuosity in these extracellular pathways should be considered [35]. Furthermore, a reduced breakdown of DA may exist due to reduced presence of metabolic enzymes for DA and/or to protection by flow in extracellular vesicles, representing another type of volume transmission [10, 36].

Furthermore, it should also be noted that very few dopamine-betahydroxylase IR terminals were found in the claustrum of the pig [13]. Nevertheless alpha 1A adrenergic receptor mRNA levels exist in the rat claustrum [37]. Further work is needed to establish catecholamine VT in the claustrum and the role of dopamine vs noradrenaline VT but the high density of D1R IR nerve cells in claustrum is impressive supporting DA VT.

Perisomatic D1R IR is found in the vast majority of the claustral nerve cell bodies and therefore the D1R IR is mainly located in the claustral glutamate projection neurons to the sensory cortices, especially layer IV. These numerous glutamate neurons dominate the claustrum [5, 38]. A DA VT signal especially in the pig likely reaches the D1R on the claustral glutamate projections. Based on the work of Liu et al. [39] it may be proposed that a D1R-NMDAR heteroreceptor complex may exist on their soma and proximal dendrites in which the D1R protomer modulates the NMDAR signaling of these claustral glutamate neurons and thus their drive to the sensory networks in the cerebral cortex. The interactions are, however, complex and can involve also PKC and PKA signaling cascades which can lead to both diminished and enhanced NMDAR protomer signaling and altered distribution of D1R in the dendritic spines [40]. The D1R monomer/homomer complexes by themselves should also have enhancing actions on the activity of the glutamate claustral-cortical neurons based on the role of the D1R in the glutamate synapses on the direct pathway from the dorsal striatum to globus pallidus interna and zona reticulata of the substantia nigra [41].

Observations were also made that the vast majority of the large glutamate claustral-cortical projection neurons also had strong kappa-opioid receptor (KOR) IR in perisomatic regions and proximal dendrites. KOR is a Gi/o coupled receptor with inhibitory effects on the AC-PKA-CREB pathway and opens up inwardly rectifying potassium channels leading to hyperpolarization [42, 43]. These events lead to inhibitory effects on neurons. The endogenous ligand for KOR is dynorphin located in the CNS [44] operating via VT [7, 10]. The primary receptor for all dynorphins is KOR for which they have a high affinity. The origin of dynorphin in claustrum is unknown but its mRNA distribution is consistent with the distribution of KOR binding sites densities [45–47]. In this way the dynorphin-KOR signal system can be regarded as an inhibitory feed-back to reduce activity in e.g., glutamate claustral-cortical pathways to the sensory cortical regions and counteract the development of salience and attention to incoming sensory inputs caused by their activation of the glutamate claustral-cortical pathways [48].

The D1R is colocated with KOR in the perisomatic membrane of the glutamate claustral-cortical pathways but there is no evidence for their heteromerization. D1R homoreceptor complexes may instead be of interest since D1R in the nucleus accumbens reward neurons enhance their reward activity [49]. It is therefore proposed that D1R mediated VT may with a delay enhance the activity of these neurons in the claustrum and enhance the salience of the sensory inputs. The neurochemical results underline the impact of integration of receptor signaling in the plasma membrane and in the intracellular cascades of the glutamate claustral-cortical neurons for counteracting salience or enhancing it.

The above view is further strengthened by the current demonstration of the existence of KOR-SSRT2 heteroreceptor complexes in the glutamate claustral-cortical neurons using the in situ PLA. We observed SSTR2 IR within distal dendritic processes of large numbers of glutamate claustral projection neurons and somatic SSTR2 IR could also be found in these neurons. With in situ PLA distinct positive clusters of 0.5–2 um in diameter were observed in relation mainly to the nerve cell bodies. Thus, integrative processes in the plasma membrane of the claustral projection neurons can also involve allosteric receptor–receptor interactions in these receptor complexes [8, 9, 50].

It should be noted that the somatostatin is released from certain claustral GABA interneurons [51] to reach via VT the SSTR2 located mainly on the distal spinal dendrites of the claustral projection neurons and mainly outside the GABA synapses. These studies further underline the importance of the inhibitory modulation of the claustral projection neurons, since the SSTR2 also is a Gi/o coupled receptor inter alia inhibiting the AC-PKA-CREB signaling pathway [52]. Multiple inhibitory mechanisms and their integration help filter out the non-salient information since by keeping a maintained low activity in the glutamate claustral projections neurons, the non-salient sensory inputs will not be noticed.

SSTR2-MOR heteromerization was previously demonstrated in cell lines with cross modulation of phosphorylation, internalization and desensitization [20]. However, the receptor–receptor interactions altering the function of the SSTR2-KOR complexes remain to be determined but SSTR2 mRNA levels was previously described in claustrum [19]. In line with the claustrum discussion above, SSTR2 activation can inhibit glutamate release and prevent status epilepticus [53] supporting the inhibitory impact of this receptor on salience development to sensory inputs.

Very few D2R IR nerve cells were found in the claustrum in the current study. It is therefore unlikely that heteromerization of SSTR2 and D2R play a significant role in the claustrum, demonstrated in cell lines using FRET and co-immunoprecipitation [54].

The immunohistochemical analysis also indicated that a subpopulation of the claustral projection neurons also expressed D4R in their cell bodies, mainly located along the medial border of the claustrum. The D4 receptors are Gi/o coupled receptors and mediate inhibitory effects of DA on neuronal activity [9, 10, 55–57]. It is therefore possible that this subpopulation of glutamate projection neurons requires an additional inhibitory mechanism to reduce their activation from sensory networks and ensure that errors in detection of salience [48] does not develop. Again short (pig) or long distance (rat) DA VT are involved in the DA D4R mediated communication.

It is probable that also this subpopulation of projection neurons possess KOR IR at the somatic level. It is therefore of interest that D4R can form heteroreceptor complexes with MOR in the striosomes [23, 58]. The existence of KOR-D4R heteroreceptor complexes should be tested for in this claustral projection system to the sensory networks and higher order association cortices using proximity ligation assay.

It is of substantial interest that a drug salvinorin A by acting at KOR produces strong synaesthesia [18]. It means e.g., that activation of vision networks leads to a somatosensory experience. There is a way to explain this phenomenon if you assume that the division of the claustrum into distinct sensory subregions is not as perfect as previously indicated [4, 59]. We propose that glutamate projections to the claustrum from the vision network can send visual cues via axons not only to the zone for vision in the claustrum but also to one more zone e.g., the somatosensory zone. Due to postulated differences in the composition of the KOR heteroreceptor complexes in the two different functional zones, salvinorin A may act as an agonist at the KOR protomer of the glutamate claustral-cortical projection neurons targeting e.g., visual cortical networks and as an antagonist at the KOR protomer of the glutamate claustral-cortical projection neurons targeting e.g., somatosensory cortical networks. As a result of the activation of the inhibitory KOR protomer the activity of the claustral-cortical glutamate projection neuron to visual networks will be blocked and no visual change will be observed. Instead the blockade induced of the KOR by salvinorin A in the glutamate claustral-cortical projection neurons targeting the somatosensory cortical networks will produce a marked increase in the activity of these projection neurons to the somatosensory cortex. A strong somatosensory experience will be obtained through the enhanced excitatory reciprocal feedback from the somatosensory cortex to the corresponding functional zone of the claustrum. This mechanism appears to explain the phenomenon of synaesthesia and underlines the impact of the changing pharmacology of heteroreceptor complexes, in this case the KOR heteroreceptor complexes, in different networks for understanding the actions of drugs on brain function.

### Endopiriform Nucleus (DEn)

It is true that the DEn has a separate development compared with claustrum [48]*.* However, the current analysis of the GPCR immunohistochemistry in the rat demonstrates a high resemblance to the corresponding features in claustrum. Thus, the density and intensity of the D1R, D2R and D4R IR nerve cell bodies was similar to what was observed in claustrum. This was true also for the KOR IR nerve cell bodies and the SSTR IR nerve cell bodies with their intensely SSTR IR distal dendritic network and the formation of the PLA positive KOR-SSTR heteroreceptor complexes.

These results are compatible with the held view that the DEn has a role for the ventrally located olfactory networks similar to the role claustrum plays for the other cortical modalities, namely to enable the decoding of the salience of the sensory inputs [48]. The major difference observed in the neurochemistry between the DEn and the claustrum was the presence of a low to moderate density of TH IR nerve terminals in the DEn vs the few scattered TH IR terminals found in the subiculum. These results may indicate a more effective dopamine volume transmission at D1Rs in the DEn, which may enhance activity in their glutamate projection neurons sending back information to the olfactory cortical neworks. Via the reciprocal excitatory feedback from the olfactory networks to the DEn the discovery of the salience of incoming olfactory stimuli may become possible [48].

In conclusion, the findings indicate that the sensory cortical glutamate drive on the glutamate claustral-cortical projection neurons is modulated by GPCRs and their receptor complexes located in the plasma membrane of these glutamate projection neurons. Their multiple DA receptors are activated by DA via long distance VT, their KOR by dynorphin proposed to be released from the claustral-cortical projection neurons themselves and their SSTR2 activated by somatostatin released from the claustral GABA interneurons forming GABA synapses on the glutamate claustral-cortical projection neurons. In this way a fine tuning of the activity of the glutamate claustral-cortical projections can be obtained from inhibition to enhancement of their signaling. They can give the sensory cortical regions significant help in deciding on the salience to be given to various incoming sensory stimuli.
